# Assessing the efficiency of exoskeletons in physical strain reduction by biomechanical simulation with AnyBody Modeling System

**DOI:** 10.1017/wtc.2021.5

**Published:** 2021-06-07

**Authors:** Lars Fritzsche, Pavel E. Galibarov, Christian Gärtner, Jonas Bornmann, Michael Damsgaard, Rudolf Wall, Benjamin Schirrmeister, Jose Gonzalez-Vargas, Daniele Pucci, Pauline Maurice, Serena Ivaldi, Jan Babič

**Affiliations:** 1 imk automotive GmbH, Ergonomics Division, Chemnitz, Germany; 2 AnyBody Technology A/S, Aalborg, Denmark; 3 Ottobock SE & Co. KGaA, Global Research, Duderstadt, Germany; 4 Istituto Italiano di Tecnologia, Center for Robotics and Intelligent Systems, Genova, Italy; 5 Université de Lorraine, CNRS, Inria, LORIA, F-54000 Nancy, France; 6 Jožef Stefan Institute, Neuromechanics and Biorobotics Lab, Ljubljana, Slovenia

**Keywords:** AnyBody Modeling System, biomechanical simulation, exoskeleton, musculoskeletal modeling, Paexo shoulder

## Abstract

**Introduction:**

Recently, many industrial exoskeletons for supporting workers in heavy physical tasks have been developed. However, the efficiency of exoskeletons with regard to physical strain reduction has not been fully proved, yet. Several laboratory and field studies have been conducted, but still more data, that cannot be obtained solely by behavioral experiments, are needed to investigate effects on the human body.

**Methods:**

This paper presents an approach to extend laboratory and field research with biomechanical simulations using the AnyBody Modeling System. Based on a dataset recorded in a laboratory experiment with 12 participants using the exoskeleton Paexo Shoulder in an overhead task, the same situation was reproduced in a virtual environment and analyzed with biomechanical simulation.

**Results:**

Simulation results indicate that the exoskeleton substantially reduces muscle activity and joint reaction forces in relevant body areas. Deltoid muscle activity and glenohumeral joint forces in the shoulder were decreased between 54 and 87%. Simultanously, no increases of muscle activity and forces in other body areas were observed.

**Discussion:**

This study demonstrates how a simulation framework could be used to evaluate changes in internal body loads as a result of wearing exoskeletons. Biomechanical simulation results widely agree with experimental measurements in the previous laboratory experiment and supplement such by providing an insight into effects on the human musculoskeletal system. They confirm that Paexo Shoulder is an effective device to reduce physical strain in overhead tasks. The framework can be extended with further parameters, allowing investigations for product design and evaluation.

## Introduction

Industrial exoskeletons are starting to become important tools for supporting workers in heavy physical tasks and un-ergonomic work conditions that are associated with high rates of absenteeism caused by musculoskeletal disorders (Fritzsche et al., 2014). Laboratory experiments and field studies are common approaches to evaluate feasibility and analyze effects of exoskeletons in industrial applications (Looze de et al., 2016) and other use cases (Settembre et al., 2020). This kind of research is necessary to get valuable and real-world insights into objective measures of physical strain (e.g., surface electromyography [sEMG], oxygen consumption [VO_2_], and motion patterns) as well as subjective comfort and user acceptance. However, laboratory experiments and field studies also have some limitations. Firstly, they require elaborate sensor technologies, such as sEMG electrodes, VO_2_ masks, and so on, which are often difficult to use and hard to implement in field research. Secondly, they do not allow conclusions about internal joint loads, stress redistribution, and compensatory mechanisms inside the human musculoskeletal system, because it is virtually not possible to measure such parameters in vivo on humans. Thirdly, studies with human participants may sometimes struggle to receive ethical approval when active devices with external power supply are tested for the first time due to safety concerns. Finally, laboratory experiments may be limited regarding the representativeness of participants and populations, not to mention to cover all possible motor deficiencies, chronic illnesses and user-specific conditions that target end-users could have. At the same time, field studies with real workers are utterly costly and may disrupt ongoing work, which often leads to a very small and nonrepresentative sample. In both cases, it is hard to systematically analyze effects on certain user populations due to a limited range and/or number of study participants.

Most of these challenges can be addressed by adding a computer simulation to the exoskeleton evaluation procedure. Sophisticated biomechanical simulation software, such as the AnyBody Modeling System (AMS), can be used to calculate effects on several parameters in relevant areas of the human musculoskeletal system, including muscle activities, joint moments, joint reaction forces, metabolism, and so on (Damsgaard et al., 2006). Although it is challenging to precisely model the physical interaction between the human body and the exoskeleton, it can provide a valuable offline tool to estimate effects of the exoskeleton on a variety of human models without any risk for the operators.

Previous research has mostly focused on building exoskeleton models in biomechanical simulations (Zhou et al., 2015), investigating the physical interaction between exoskeletons and the human body (Spada et al., 2019) or comparing different options for mechanical design (Zhou et al., 2017; Bornmann et al., 2020), but less on computing biomechanical effects in simulated work activities conducted by human subjects supported by commercial exoskeletons. This study applies the biomechanical simulation framework of AMS using a large set of motion capturing data recorded at a laboratory experiment applying the commercial exoskeleton Paexo Shoulder in an overhead drilling task and evaluates simulation results in the scope of previous experimental outcomes. Hence, this study not only quantifies the simulated effects of the specific exoskeleton to reduce physical strain, but also helps to evaluate reliability and validity of the simulation framework by comparing simulation results with physiological measures (sEMG activity, VO_2_ consumption, and heart rate) and with subjective ratings (NASA TLX questionnaire) that were measured during the original laboratory experiment.

## Material and Methods

### Data set from the laboratory experiment

**Figure 1.**
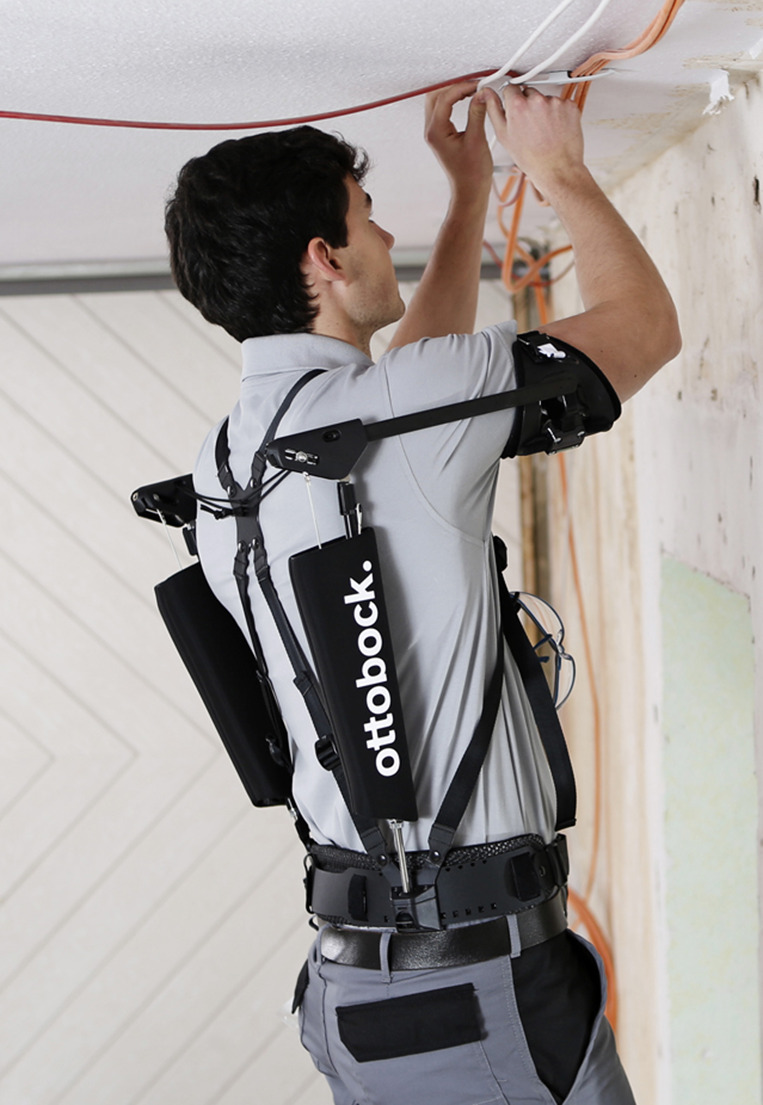
Paexo Shoulder exoskeleton used in construction industry.

The biomechanical simulation is based on data of the laboratory experiment presented in Maurice et al. (2020). The dataset is available on Zenodo (Maurice et al., 2018). In the original study, 12 participants performed an overhead drilling task with a hand-held tool (0.66 kg). The tool was always used with the right hand, whereas the left hand was only needed to stabilize the body. Participants had to point the tool as fast as possible from one point to a target point, both located on a horizontal screen above the participant’s head, and remain at the target. All participants performed the task wearing the Paexo Shoulder exoskeleton (WE), and without wearing it (NE) in randomized order. Paexo Shoulder is a commercially available passive exoskeleton (i.e., without external power source) with a weight of 1.9 kg provided by Ottobock SE & Co. KGaA, Duderstadt, Germany. It is specifically designed to support static and dynamic overhead work (Figure 1).

**Figure 2.**
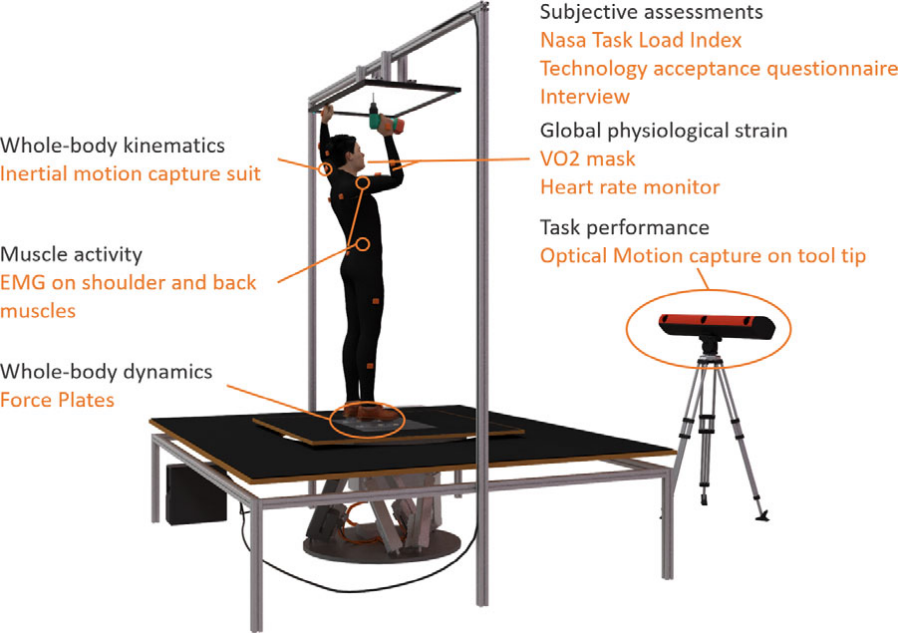
Experimental set up for data recording at the laboratory experiment.

The experimental set up at the laboratory included a variety of sensor technologies (Figure 2). Whole-body kinematics were retrieved using an inertial motion tracking suit consisting of 17 inertial measurement units (Xsens MVN Awinda from Xsens Technologies, Enschede, The Netherlands). Data were recorded over the whole movement sequence of a trial with the Xsens MVN Analyze software version 2018.0.0 at 60 Hz. The system was calibrated following the standard Xsens MVN calibration procedure. Data of each trial and participant were later transferred to the simulation software and used for the biomechanical analysis. Additionally, muscle activity of the right anterior deltoid and right erector spinae longissimus was recorded with the Biometrics DataLOGMW8X EMG system using the SX230 sensor. The heart rate was measured using a Polar WearLink+ Transmitter with Bluetooth sensor and the oxygen consumption was measured with a VO_2_ Master Pro mask.

### Simulating overhead work with Paexo Shoulder

Simulation of the overhead task was done with the AMS v.7.3.2 provided by AnyBody Technology A/S, Denmark. AMS is an inverse dynamics simulation platform for biomechanical analysis of the human body subjected to a physical activity and to interaction with elements of the environment. Simulation in AMS uses a state-of-the-art human body model, constructed on data from detailed cadaveric dissection studies and/or defined based on anatomy textbooks (Damsgaard et al., 2006). The human model is comprised of most of the muscle elements, bones, and joints in the human body. The system computes muscle activities, joint moments, and reactions forces necessary to generate specified motion and counterbalances applied external forces by recruiting muscles in an optimal way (Rasmussen et al., 2001).

**Figure 3.**
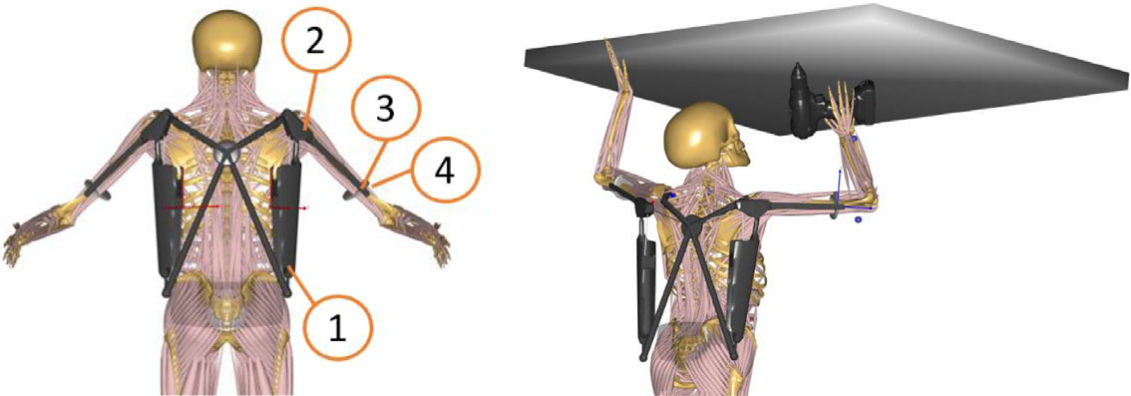
AnyBody Model with Paexo Shoulder exoskeleton (left); application in overhead task (right).

In the first step, a rigid body dynamics model replicating the actual design of Paexo Shoulder exoskeleton was created (Figure 3). Computer-Aided Design (CAD) drawings and detailed information about joint positions were provided by Ottobock to construct an accurate inverse dynamics model. The right and left sides were modeled symmetrically. Each side consisted of (a) a back component attached to the socket on the belt allowing free rotation in all directions, (b) an arm component attached to the back part through a revolute joint, (c) a cuff attached through a revolute joint to the arm bar, and (d) a prismatic joint to the arm of the subject.

The modeled points of contact with the human body are the following: the belt of the PAEXO is attached to the pelvis with a spherical joint and the cuff on the arm bar of the PAEXO is attached to the humerus with a prismatic joint. Additional soft kinematic constraints were imposed to allow minor rotation of the cuff and violation of the rigid linkage of the cuff joint to simulate soft tissue deformations of the upper arm and shoulder regions. The load transfer between human and exoskeleton including its weight of 1.9 kg was modeled as linear reaction forces in the spherical joints, assuming friction-free rotation of the rods in the socket for the back-belt interface and as linear reaction forces to the humerus as an approximation for the cuff-humerus interfaces. The muscle recruitment optimization procedure computes necessary reaction forces to provide dynamic equilibrium for relevant bodies. The shoulder support torque of Paexo Shoulder was modeled as a function of shoulder angle with the data provided by Ottobock.

To simulate the overhead drilling task, motion capture datasets from the laboratory experiment were processed using a dedicated motion capture processing model in the AnyBody Managed Model Repository (Lund et al., 2019). Each recording was subdivided into 5 sets of 24 trials according to the experimental protocol. Each trial consisted of one pointing motion, staying at the target position for approx. 2 s, and going back to the starting point (the total trial duration was approx. 3 s). The Python-based library AnyPyTools (Lund M et al., 2019) was applied to batch-process a total of 2.880 trials (12 participants, 2 conditions, 5 sets of 24 trials). The processing model used anthropometric measurements available in the Xsens .bvh files to scale corresponding model body parts to represent body size of the participants. Once the model was adjusted anthropometrically, the human model motion was generated through an optimization procedure that established correspondence with the joint angles of the Xsens avatar. Finally, inverse dynamics analysis using optimization for muscle recruitment was carried out to compute muscle activities and joint reaction forces.

### Data analysis

Based on the repeated-measures design of the laboratory experiment (i.e., each participant conducted the overhead drilling task with and without exoskeleton in randomized order), simulation data were grouped into two conditions “with exoskeleton” (WE) and “without exoskeleton” (NE). The difference between the two conditions (noted as ∆NE–WE) is calculated for various variables with the purpose of assessing the influence of the exoskeleton. Data analysis was performed over the whole movement sequence of a trial, including both the dynamic and static part of the trial, but excluding resting periods between trials**.** Descriptive data analysis included calculating box-plots and histograms for each variable. Single outlier values beyond three standard deviations (SDs) above or below the mean were excluded for each variable. Wilcoxon signed-rank test was used to test for significant differences between the two conditions, since most of the data was not normally distributed according to Kolmogorov–Smirnov test. Results were considered as statistically significant with *α* < 0.05 (two-sided). Data analysis was also done using a script written in Python code to automatically analyze the 2.880 trials for each simulation variable. The Wilcoxon function from SciPy.stats-package was applied for statistical analysis and the boxplot function from Matplotlib.Pyplot-package was applied for data visualization.

*Muscle activity* is measured as a percentage of the estimated maximum activation of each muscle according to the AnyBody model. With regard to muscle activities, the most relevant body area is the shoulder with its deltoid muscles (anterior, lateral, and posterior). Other muscles in the shoulder/arm area which are considered relevant are the trapezius (descendant part), infraspinatus, supraspinatus, biceps brachii, and triceps brachii muscle. Regarding the chest and back regions, the pectoralis major and the lumbar part of erector spinae muscle were analyzed. *Joint reaction forces* were mainly analyzed for relevant joints in the shoulder/arm area, more precisely the glenohumeral, acromioclavicular, and sternoclavicular joints. *Compression forces* were analyzed for the lumbar spine area, more precisely between the L5 and S1 disc area. All definitions and names of joint reaction forces are in accordance with International Socitey of Biomechanics (ISB) recommendations: Part 1 for ankle, hip, and spine (Wu et al., 2002); Part 2 for shoulder, elbow, wrist, and hand (Wu et al., 2005).

## Results

Biomechanical analysis focused on body areas that are most relevant for the overhead task in the experiment and areas with possible adverse side effects. Simulation results are presented in three sections: (a) muscle activities in arm, shoulder, and back area, (b) joint reaction forces in arm/shoulder area, and (c) compression forces in the lumbar spine and the hip.

### Muscle activity

**Figure 4.**
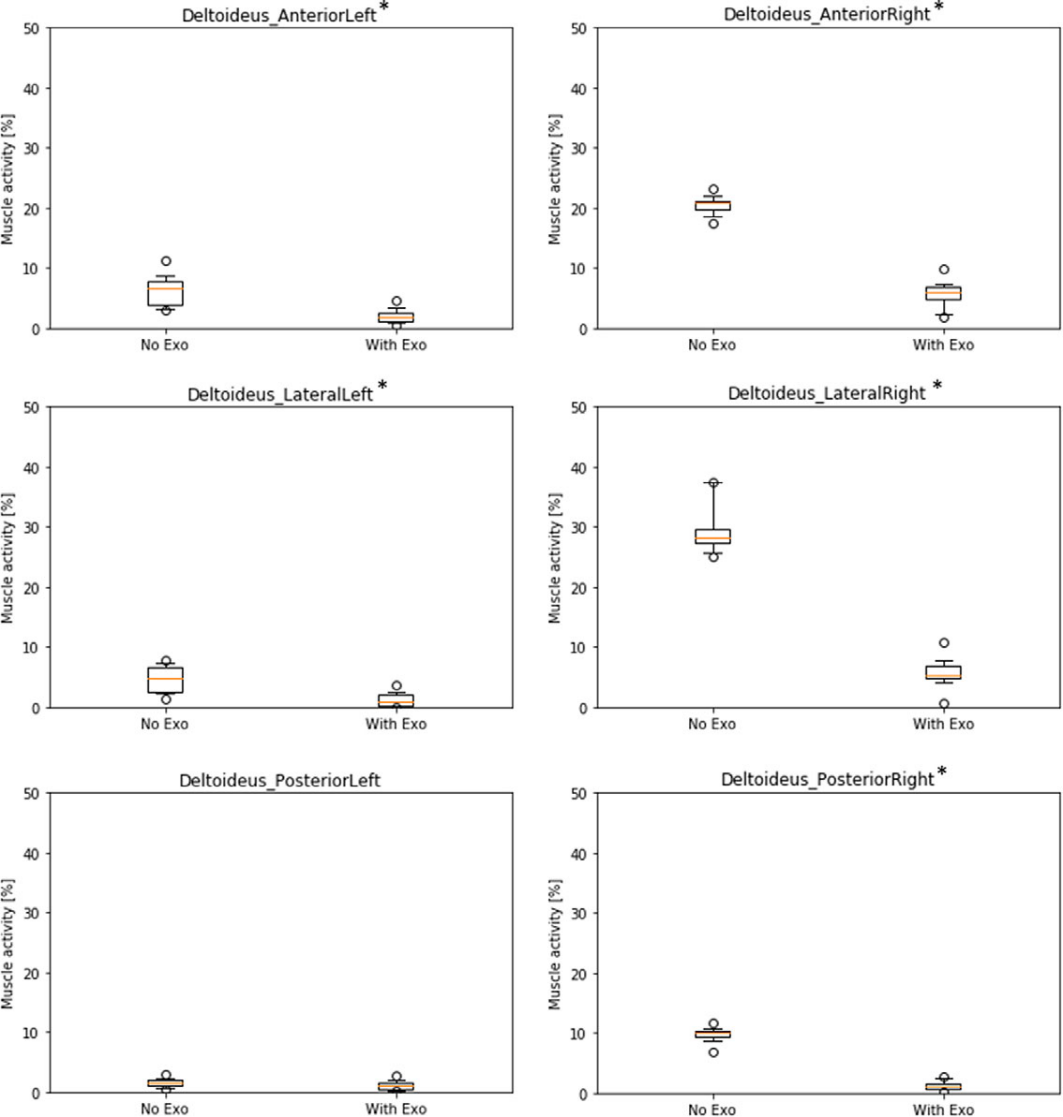
Boxplots of simulated muscle activity (% of maximum activation) of left and right deltoid muscles comparing conditions without (NE) and with (WE) exoskeleton (* shows significant differences, *p* < .01).

Muscle activity was calculated for relevant shoulder/arm muscles and the back. Figure 4 shows box plots for the left and right shoulder deltoid muscles. The left body side, which was not actively involved in the drilling task, generally shows a lower muscle activity (below 10% for all deltoid muscles in both conditions). Nevertheless, differences between NE and WE conditions are significant for anterior (∆NE–WE = 4.3%, *p* < .01) and lateral (∆NE–WE = 3.4%, *p* < .01) deltoid at the left body side indicating a lower muscle activity in the condition with exoskeleton, but not significant for posterior (∆NE–WE = 0.5%, *p* = .18) deltoid. The muscles of the right body side were involved in arm movements and were holding the drill with a weight of 0.66 kg and therefore showed higher activity patterns. Absolute differences in muscle activity between NE and WE conditions are significant for all three deltoid muscles (anterior: ∆NE–WE = 14.9%, *p* < .01; lateral: ∆NE–WE = 23.7%, *p* < .01; posterior: ∆NE–WE = 8.5%, *p* = <.01). Compared to the mean activity across all subjects and all trials in NE condition as baseline, the relative reduction of muscle activity in the WE condition is considerably high: 73.6% for anterior, 81.8% for lateral, and 87.4% for posterior deltoid.

**Figure 5.**
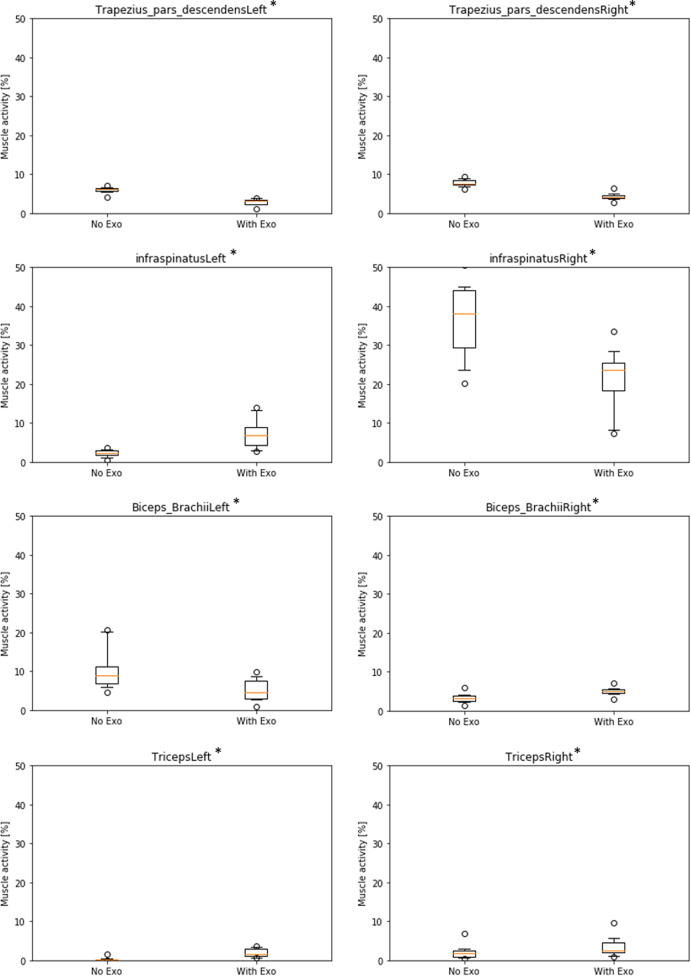
Boxplots of simulated muscle activity (% of maximum activation) of left and right shoulder/arm muscles comparing conditions without (NE) and with (WE) exoskeleton. (* all significant with *p* < .01).

In addition to deltoid, some other relevant muscles in the shoulder/arm area were analyzed (Figure 5). For the trapezius muscle, differences between NE and WE were significant for the left side (∆NE–WE = 3.1%, *p* < .01) and for the right side (∆NE–WE = 3.5%, *p* < .01), indicating a lower muscle activity in the WE condition. Results for infraspinatus muscle were divergent: they showed a significant increase of muscle activity on the left side (∆NE–WE = −4.9%, *p* < .01) and a decrease of muscle activity on the right side (∆NE–WE = 15.1%, *p* < .01) in the WE condition with relatively high baseline activity in the NE condition (37% on average). In the opposite, the biceps brachii muscle showed a significant decrease of activity on the left side (∆NE–WE = 5.2%, *p* < .01) and a small increase on the right side (∆NE–WE = −1.8%, *p* < .01) in the WE condition. Finally, the triceps brachii muscle showed a small but significant increase of muscle activity on both left side (∆NE–WE = −1.8%, *p* < .01) and right side (∆NE–WE = −1.5%, *p* < .01) in the WE condition. In summary, all these muscles (except infraspinatus) showed very low activity patterns with only small differences between conditions that may not be relevant in practice.

Furthermore, simulation results for other shoulder muscles like supraspinatus and pectoralis did not show any significant differences (with *p* < .05) of activity patterns between NE/WE conditions at all. More importantly, there were also no significant differences found between NE/WE conditions in lumbar erector spinae muscles, with medium activity (ca. 20% in average) for both left and right side (not illustrated).

### Joint reaction forces

Joint reaction forces results are only reported for the right body side. Results for the left side are not reported because, similar to muscle activities, they are much smaller when compared to the right side since the overhead drilling tool was used with the right hand only.

**Figure 6.**
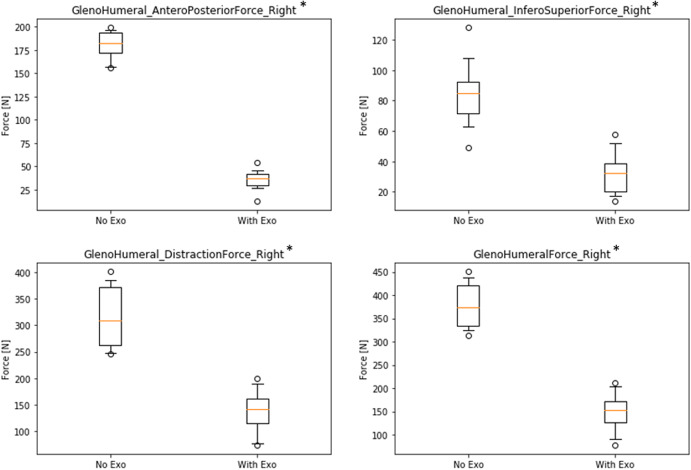
Boxplots of simulated forces of the right glenohumeral joint in three directions comparing conditions without (NE) and with (WE) exoskeleton (* all significant with *p* < .01).

Figure 6 shows results for the glenohumeral joint indicating a decrease of forces in the WE condition for all three force directions and in the total resulting force in absolute and relative values (antero-posterior: ∆NE–WE = 145.1 N, rel.∆ = 80.3%; infero-superior: ∆NE–WE = 51.9 N, rel.∆ = 61.7%; distraction: ∆NE–WE = 176.9 N, rel.∆ = 55.9%; Total: ∆NE–WE = 229.2 N, rel.∆ = 60.5%; all significant with *p* < .01).

**Figure 7.**
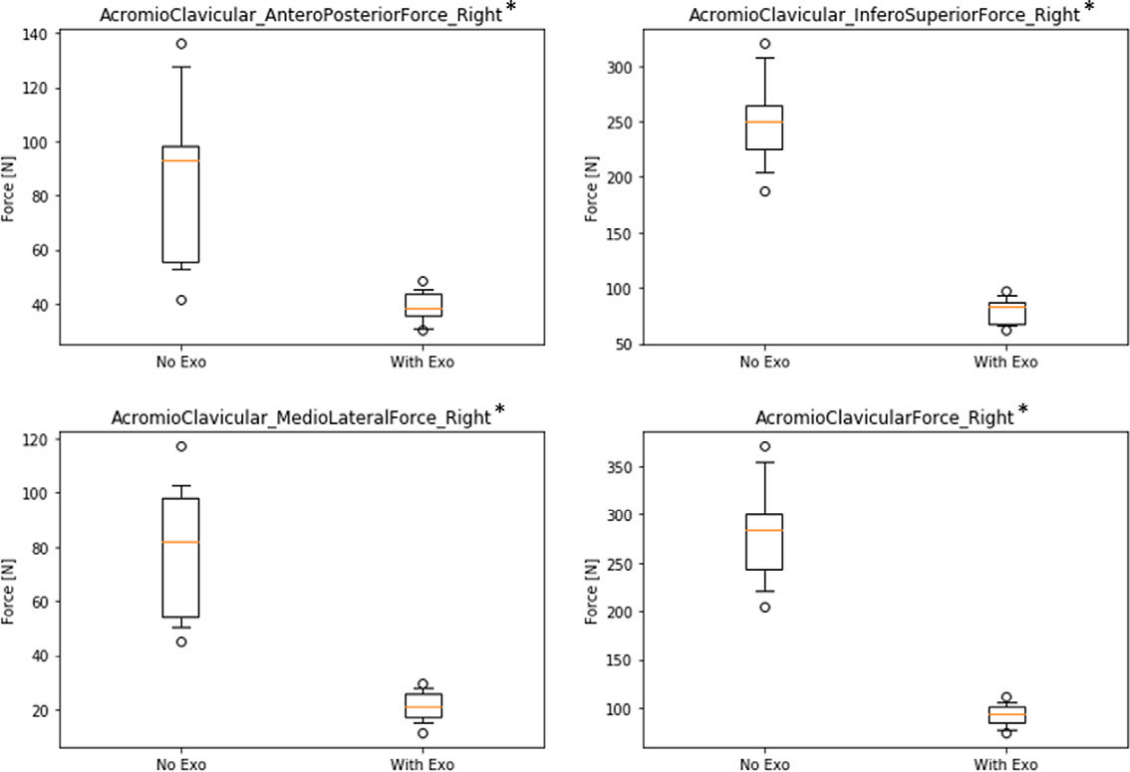
Boxplots of simulated forces of the right acromioclavicular joint in three directions comparing conditions without (NE) and with (WE) exoskeleton (* all significant with *p* < .01).

Likewise, Figure 7 shows results for the acromioclavicular joint indicating a decrease of forces in the WE condition for all three force directions and in the total resulting force in absolute and relative values (antero-posterior: ∆NE–WE = 46.0 N, rel.∆ = 54.1%; infero-superior: ∆NE–WE = 170.9 N, rel.∆ = 68.3%; medio-lateral: ∆NE–WE = 58.0 N, rel.∆ = 73.2%; Total: ∆NE–WE = 189.2 N, rel.∆ = 67.1%; all significant with *p* < .01).

**Figure 8.**
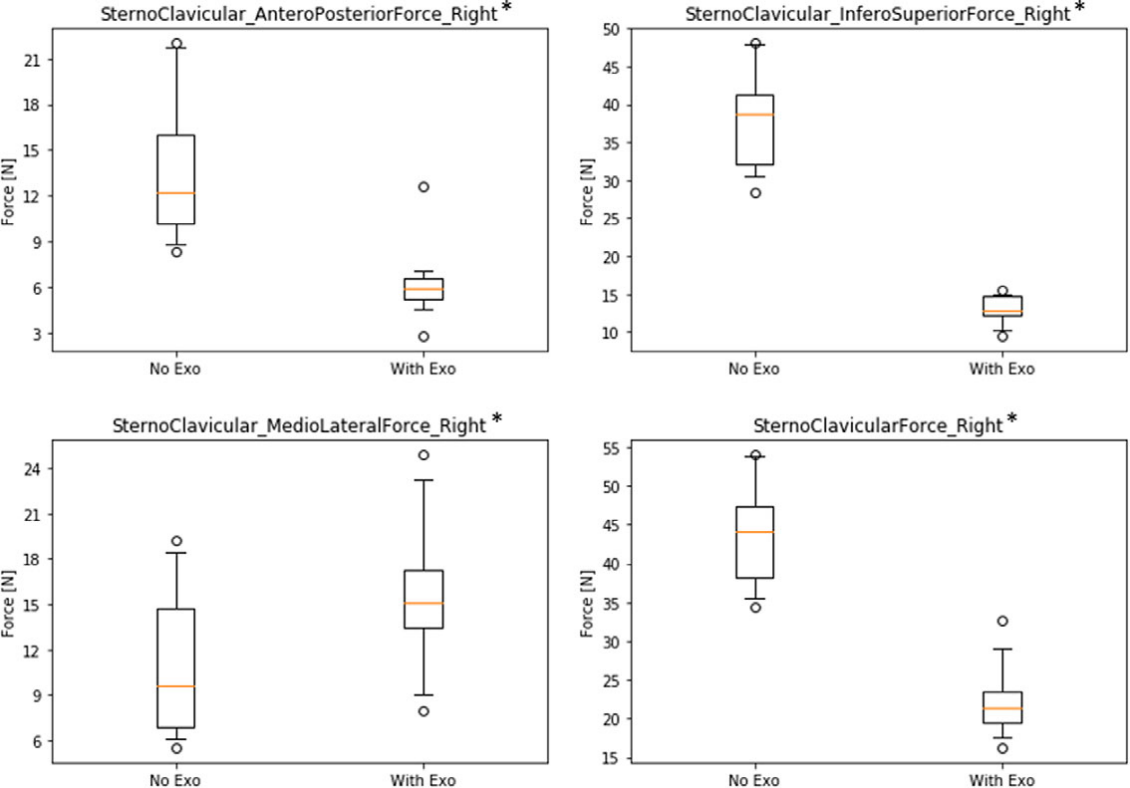
Boxplots of simulated forces of the right sternoclavicular joint in three directions comparing conditions without (NE) and with (WE) exoskeleton (* all significant with *p* < .01).

Figure 8 shows results for the sternoclavicular joint. Absolute force values are quite low compared to the other two joints with a maximum of *F* = 43.4 N (SD = 6.6 N) in the total resulting force for the NE condition. Two force directions and the total resulting force show the same pattern like the other two joints indicating a decrease of forces in the WE condition in absolute and relative values (antero-posterior: ∆NE–WE = 7.4 N, rel.∆ = 54.4%; infero-superior: ∆NE–WE = 24.9 N, rel.∆ = 65.9%; Total: ∆NE–WE = 21.1 N, rel.∆ = 48.5%; all significant with *p* < .01). However, there is an opposite effect in the medio-lateral force direction (∆NE–WE = −4.5 N), but on a very low force level (NE: *M* = 11.0 N, SD = 4.8 N; WE: *M* = 15.5 N, SD = 5.0 N).

**Figure 9.**
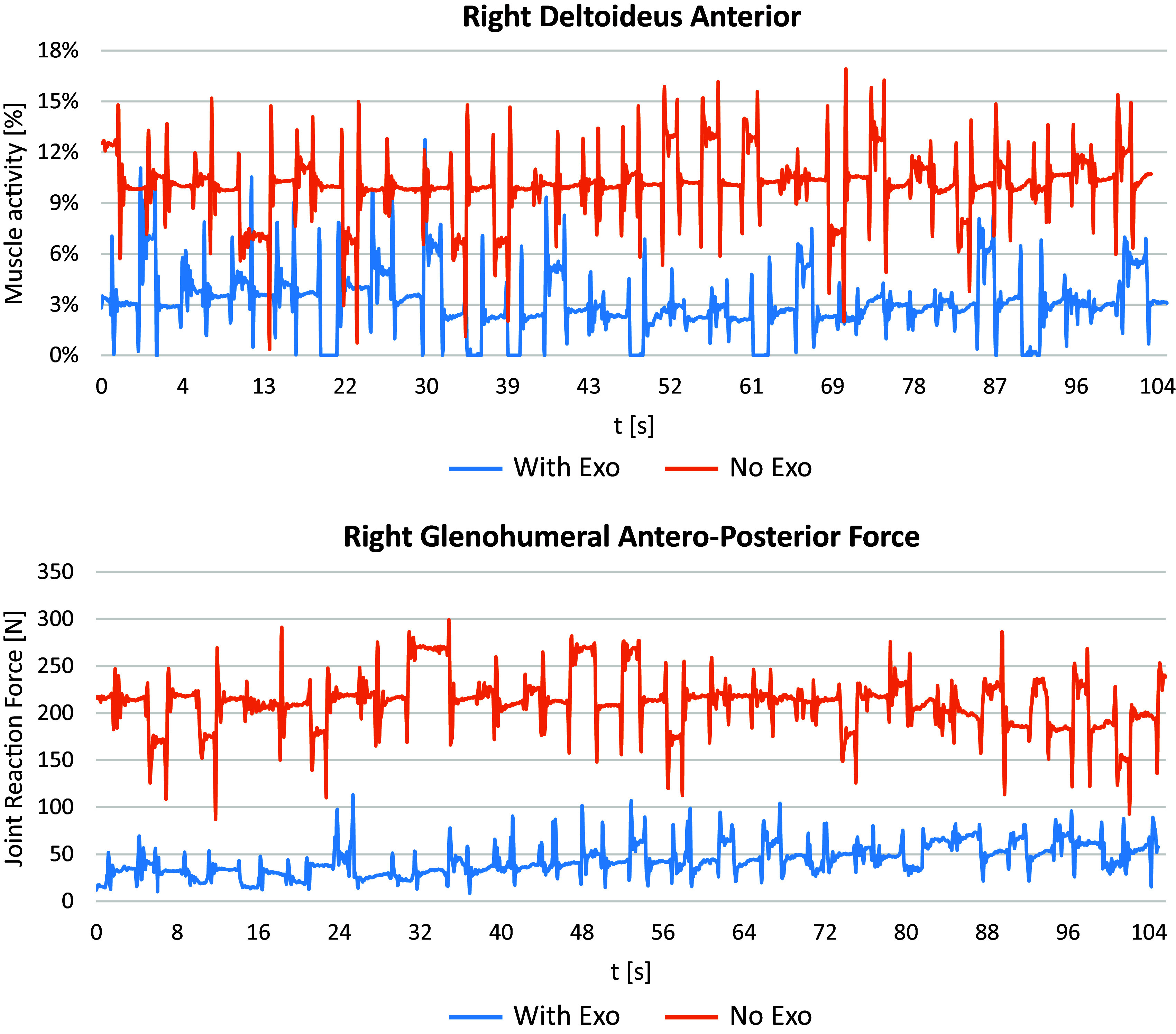
Example of right deltoid muscle activity and glenohumeral antero-posterior force across time for one trial with exoskeleton and one trial without exoskeleton of a single participant (subject #6).

In order to illustrate the differences in muscle activity and joint reaction force across time between the two conditions, Figure 9 shows an example for one trial with exoskeleton and one trial without exoskeleton of a single participant. During the time of the trial of approx. 105 s, the muscle activity of the right deltoid anterior is almost constantly lower in the trial with exoskeleton as compared to the trial without exoskeleton. Differences are even clearer for the glenohumeral antero-posterior force, ranging between 8.4 N and 113.2 N for the trial with exoskeleton compared to 87.1 N and 299.1 N for the trial without exoskeleton.

### Compression forces

**Figure 10.**
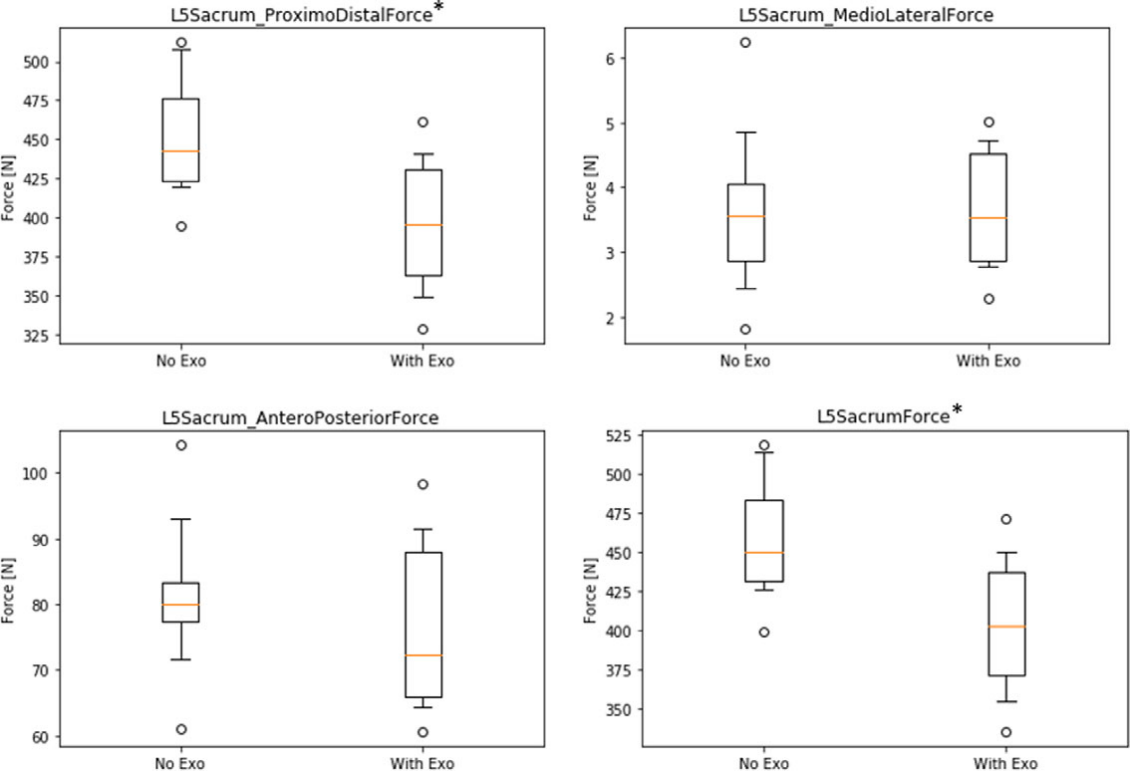
Boxplots of simulated forces in the L5/S1 disc area in three directions comparing conditions without (NE) and with (WE) exoskeleton (* indicates significant differences with *p* < .01).

Compression forces of the L5/S1 disc are shown in Figure 10, indicating a significant decrease in proximo-distal direction (∆NE–WE = 56.1 N, rel.∆ = 12.4%, *p* < .01) and in total resulting force (∆NE–WE = 56.0 N, rel.∆ = 12.2%, *p* < .01) in the WE condition. No significant differences were observed in antero-posterior (∆NE–WE = 4.5 N) and medio-lateral (∆NE–WE = 0.0 N) direction. Similarly, forces in the hip showed no significant difference in most parameters, except for a small decrease in antero-posterior direction (left side: ∆NE–WE = 4.0 N, rel.∆ = 17.3%, *p* < .05; right side: ∆NE–WE = 12.4 N, rel.∆ = 37.3%, *p* < .05) in the WE condition (not illustrated).

## Discussion

Based on data from 12 participants and more than 2,800 trials recorded in a laboratory experiment with Xsens MVN motion capturing suit, this study investigated the biomechanical effects of wearing the Paexo Shoulder exoskeleton for supporting an overhead drilling task using musculoskeletal simulations carried out with AMS. The major findings are discussed with focus on (a) the effects on muscle activity and joint reaction forces in order to evaluate the efficiency of the exoskeleton to support the work task and (b) the comparison of simulation results with previous outcomes of the laboratory study in order to investigate similarities and differences between simulation and experimental measurements.

### Exoskeleton effects on muscle activity and joint reaction force

Simulation results indicate that the exoskeleton strongly reduces activity of relevant shoulder muscles. Comparing the two experimental conditions of conducting the overhead task with/without exoskeleton shows that muscle activity is reduced when wearing the exoskeleton between 9 and 24% in absolute numbers, corresponding a relative decrease of 74–87% for the right-side deltoid muscles as primary actuators for the right-handed overhead drilling task. The left-side shoulder generally shows lower muscle activity (below 10%), because it is only stabilizing the body during the overhead task. However, left arms are also held in an overhead position, so the small but significant decrease in deltoid muscle activity (1–4%) suggests, that the exoskeleton is also supporting the nondominant body side to some extent. Results also demonstrated that other relevant muscles in the shoulder/arm area had very low activity patterns, with only one exception. Infraspinatus muscle showed a quite strong baseline activity at the right side (37% without exoskeleton) with a medium decrease of 15% with exoskeleton. On the contrary, at the left side it showed a small increase of muscle activity of 5% on a much lower baseline (below 5%). In this case, the exoskeleton supports the body side that is actively conducting the overhead task but it does not help the left body side. Additionally, during exoskeleton use minor decreases of muscle activity were observed for trapezius and biceps brachii left side, while small increases of muscle activity were seen for biceps and triceps brachii right side. Considering the very low baseline activity of these muscles (all below 10%) and the small changes (all below 5%), these results can be considered as not practically relevant.

Overall, simulated muscle activity with and without exoskeleton for left and right side shows a very plausible pattern of results. Muscles that are generally involved in the active task on the right-side arm/shoulder area show the highest activity patterns in both conditions and they also benefit most from the use of the exoskeleton, particularly deltoid muscles and infraspinatus. The activity of all three deltoid muscles is reduced by more than 70% at the right side and average muscle activity is reduced to below 10% on both left and right side. This can be considered as practically relevant and may contribute to the prevention of musculoskeletal disorders in the shoulder because muscle activity is consistently below the 15%-threshold of the recommended permanent endurance limit according to Rohmert (1986) only while wearing the exoskeleton. While this accounts for deltoid muscles, infraspinatus was also strongly activated by the overhead task and is still above the 15%-threshold while wearing the exoskeleton. The relative gain of exoskeleton use is not as strong as for the deltoid muscles. This could be a target for further optimization of the exoskeleton: the manufacturer should try to improve the design in order to reduce activity in infraspinatus muscle further and keep it below the 15%-threshold.

Similar to muscle activity, the use of the exoskeleton also strongly reduces reaction forces in relevant shoulder joints. Looking only at the active right-side shoulder, results showed a decrease in the glenohumeral joint while wearing the exoskeleton for all three force directions between 56 and 80% with 229 N absolute difference for the total resulting force. Likewise, forces in the acromioclavicular joint are reduced for all three force directions between 54 and 68% with 189 N absolute difference for the total resulting force. In addition, forces in the sternoclavicular joint are reduced in two directions between 54 and 66% with only 21 N absolute difference for the total resulting force. There is one very small deviation showing an opposite effect in the medio-lateral force direction of the sternoclavicular joint with an increase of 5 N, which is negligible compared to all other results. Thus, overall results are very consistent across different joints and force directions indicating that the exoskeleton is actually doing what it is supposed to do: substantially reduce physical strain in the shoulder joints with almost no opposing effects.

In order to find adverse side effects in other areas of the body, this study investigated muscle activity and compression forces in the lower back and the hip. Results showed a medium activity level in erector spinae muscles on left and right side (ca. 20%), but there were no differences WE/NE use. Moreover, compression forces in the L5/S1 disc area were unchanged in two directions or even slightly decreased (ca. 12%) while wearing the exoskeleton. Similarly, forces in the hip showed no significant difference in most parameters, except for a small decrease in one force direction. Overall, muscle activity and joint forces in the lower back and the hip were either unchanged or even decreased using the exoskeleton. These results are suggesting that no adverse side effects appeared and that the re-distribution of forces from the shoulder to the lower body works very well with the Paexo Shoulder exoskeleton.

### Comparison of simulation results and laboratory measurements

In order to evaluate the possibility to use simulation results from AMS as supplement for experimental studies with human participants during the development and testing of exoskeletons, this study also compared simulation results with results from the previous laboratory study (detailed description in Maurice et al., 2020). In the lab, sEMG measurements were only recorded for two muscles: anterior deltoid muscle (front shoulder) and erector spinae muscle (along the spine). Hence, the comparison can only be made with regard to these two variables. For erector spinae, both lab and simulation results do not show any difference between WE/NE conditions. So they align well. For anterior deltoid, the sEMG activity was decreased by 54% on average while wearing the exoskeleton, whereas the same muscle activity is reduced by 74% in the simulation model. Both results are showing a strong beneficial effect of the exoskeleton reducing muscle activity in the intended area of the body, so they support each other quite well. The effect that is predicted by the simulation is a bit stronger than the measurement on human participants. This may be explained by the fact that the simulation is more specific in calculating activities of separate muscles and even muscle parts using the underlying assumptions of load distribution in the AMS model (Rasmussen et al., 2001; Damsgaard et al., 2006). This allows to differentiate the involvement of single muscles in a specific activity. In comparison, sEMG measurements are less specific because they can be influenced by occurring cross-talk between muscles and depend on the position of the electrodes with respect to the muscle innervation zone. This can be problematic in dynamic situations, such as the work task in the lab experiment, where innervation zones may move under the sEMG electrodes, which may be misinterpreted as changes of muscle activity level (Lund et al., 2012). So the recorded activity at the skin surface is often influenced by more than just one specific muscle, even if electrodes are placed very precisely at the beginning of the experiment.

Furthermore, many other outcome parameters of the simulation could not be compared at all, because they were not measured in the lab but only computed during simulation. It would require a lot more EMG measurement points and a huge amount of extra effort in sEMG measurement to fully compare simulation and lab results. This is actually a strong advantage of the simulation in AMS: once it has been set-up, it can compute many outcome variables at the same time without extra effort and later be revisited for extra output variables if needed while ensuring repeatability of the experiment. It may also be used for investigating muscles that are not close to the skin surface and thus, can hardly be measured with sEMG. In summary, simulation results might be more detailed, more precise and less prone to uncontrolled perturbation variables but they may also be quite idealistic because they assume optimal conditions of use.

In general, it should be considered that interpretation of simulations requires establishing correspondence between output of simulations and physiological processes. However, this correspondence is not always feasible. Muscle activities computed in simulations represent a percentage of a maximum muscle force needed to perform selected motion and do not necessarily match sEMG signal on absolute scale (Lund et al., 2012): sEMG signals are typically very individual and therefore need re-reprocessing and normalization in order to remove artefacts and make measurements comparable between participants. This was also done for the measurements in the lab (Maurice et al., 2020). The AMS model uses a built-in normalization based on the estimated/calculated maximum of muscle force needed to conduct a certain activity (Rasmussen et al., 2001; Damsgaard et al., 2006). Therefore, direct comparison between simulated muscle activity and sEMG signals should be made with caution, which is why this study focused on comparing trends and relative changes in the two experimental conditions instead of looking at absolute values.

Finally, it should be mentioned that the lab study also included the measurement of metabolic parameters, such as oxygen consumption (decreased by 33% with exoskeleton use) and heart rate (decreased by 19% with exoskeleton use), as well as subjective ratings on comfort and effort (decreased by 21% in overall effort with exoskeleton use) from the participants. Although metabolic calculations for the entire body are possible with the AMS simulation, they were not part of this study and will be analyzed in future studies.

Obviously, simulation studies cannot replace subjective feedback on comfort and effort from human participants. However, this is still very important for designing human-centered wearable devices and reaching a high level of acceptance in field applications. Therefore, the authors clearly propose to use simulation studies as a supplemental approach that provides further insights about biomechanical effects inside the human body, rather than pledging to completely replace studies with human participants.

Overall, comparing simulation results and laboratory measurements based on the available data suggests that outcomes align quite well despite many theoretical and practical differences between both approaches. This indicates that elaborate biomechanical simulations, such as the one presented in this study, can be very beneficial for exploring the impact of exoskeletons or other wearable devices on the musculoskeletal system. More specifically, they can be used to evaluate changes in internal body loads as a result of wearing exoskeletons and thus, supplement laboratory experiments and field studies by providing an insight into effects on the inside of the human body. In this regard, they can be even more detailed and may be more precise than standard sEMG measurements. Thus, simulations can provide developers and manufacturers of exoskeletons valuable indications on how the device would affect end users. They can also be applied to iteratively optimize a device before having to carry out real experiments with humans, so they may finally help to speed up development and testing of new products. In addition, results obtained from biomechanical simulations can be utilized to precisely define the hypothesis and assumptions for validation tests with actual users in laboratory and field studies, which would simplify and strengthen the experimental design and focus the test on the most relevant questions. Studies can be made more efficiently, potentially saving costs for implementation and accelerating time-to-market.

### Limitations and future research

Methodological limitations are common and inherited from the conventional musculoskeletal modeling approach, such as assumptions of a particular muscle recruitment, representation of anatomy by mechanical elements (rigid bodies, springs, massless actuators, etc.), and so on. These limitations are commonly used for biomechanical simulations, and deem to be acceptable for this study. Limitations in the simulation design were dictated by the lack of experimental data: (a) motion of the exoskeleton was computed according to the kinematic model since it was not measured in the experiment, (b) virtual fitting of the exoskeleton was done for each subject according to the manufacturer’s guidelines and may contain some discrepancies with reality. However, the fitting data were reviewed by experts from Ottobock, the exoskeleton manufacturer, and was considered to be accurately representing exoskeleton behavior. Another general limitation of simulations is the assumption of ideal and stable conditions throughout the use of the device with regard to fitment to the human body and surrounding conditions. In reality, some conditions might change over the course of usage: the fitment to the body might get displaced, the participants start to sweat, and so on. As explained above, this may lead to differences between simulation results and real-life effects for exoskeleton users, but it also allows to determine the potential effects (positive and negative) of exoskeletons without perturbation of uncontrolled variables.

Future research should take into account that the present study and the simulation framework were developed based on the evaluation of passive exoskeletons with mechanical components (springs, etc.) and without external power supply. It seems more difficult to apply for soft exoskeletons that mainly use textile straps and structures for providing support in certain awkward postures and/or load handling. The framework also needs to be extended for the evaluation of active exoskeletons that augment user capabilities with external power sources. In this case, the control mechanisms need to be modeled very carefully and added to the simulation. Another field of future research is the question how biomechanical simulations can be prepared with very few or even without any motion capturing data. AMS already allows to do that using AnyScripts, but it still requires a lot of expertise and effort to create realistic biomechanical simulations. Other digital human models, such as ema Work Designer (Bauer et al., 2019), use sophisticated algorithms for generating artificial motions in work situations that can be created with less effort and potentially could be used as an input for AnyBody simulations (Peters et al., 2019). Using artificial motions as input for biomechanical simulations would make such studies independent from laboratory recordings and allow investigations of virtual exoskeleton prototypes. However, further developments and validation studies are needed to finally reach this goal.

## Conclusion

This study demonstrates how a simulation framework could be used to evaluate changes in the internal body loads as a result of wearing exoskeletons. Simulation results agree with experimental measurements and supplement such by providing an insight into effects on the changes in the human musculoskeletal system. In future research, the framework can be extended by analyzing a variety of basic movements with different human populations and more biomechanical parameters. It allows investigating intended main effects as well as side effects of exoskeletons and possibly other wearable devices. Such analysis can not only be used for product evaluation, but also during the development and design stage of new products.

Specific results of the study suggest that Paexo Shoulder is an effective device to reduce physical strain in overhead tasks. Muscle activities of the shoulder complex are reduced, which should decrease the fatigue level of the worker. Similarly, it reduces joint reaction forces in the shoulder supposedly leading to a decrease in shoulder joint cartilage degeneration rates. Moreover, the device does not redistribute the arm loads onto the lumbar spine, indicating that no adverse side effects for the lower back area have to be expected. Comparison with measured muscle activity and physiological parameters shows that simulation results are quite similar, underlining the effectiveness of the exoskeleton in reducing overall strain.

## Data Availability

As stated in the text, study data is available at Zenodo: http://doi.org/10.5281/zenodo.1472214. For further information, please contact the corresponding author.
